# The Metabolomic Profile of Umbilical Cord Blood in Neonatal Hypoxic Ischaemic Encephalopathy

**DOI:** 10.1371/journal.pone.0050520

**Published:** 2012-12-05

**Authors:** Brian H. Walsh, David I. Broadhurst, Rupasri Mandal, David S. Wishart, Geraldine B. Boylan, Louise C. Kenny, Deirdre M. Murray

**Affiliations:** 1 Neonatal Brain Research Group, Department of Paediatrics and Child Health, Cork University Maternity Hospital, Wilton, Cork, Ireland; 2 Department of Medicine, University of Alberta, Edmonton, Alberta, Canada; 3 Departments of Biological and Computing Sciences, University of Alberta, Edmonton, Alberta, Canada; 4 Department of Obstetrics and Gynaecology, University College Cork, Cork, Ireland; Hôpital Robert Debré, France

## Abstract

**Background:**

Hypoxic ischaemic encephalopathy (HIE) in newborns can cause significant long-term neurological disability. The insult is a complex injury characterised by energy failure and disruption of cellular homeostasis, leading to mitochondrial damage. The importance of individual metabolic pathways, and their interaction in the disease process is not fully understood. The aim of this study was to describe and quantify the metabolomic profile of umbilical cord blood samples in a carefully defined population of full-term infants with HIE.

**Methods and Findings:**

The injury severity was defined using both the modified Sarnat score and continuous multichannel electroencephalogram. Using these classification systems, our population was divided into those with confirmed HIE (n = 31), asphyxiated infants without encephalopathy (n = 40) and matched controls (n = 71). All had umbilical cord blood drawn and biobanked at −80°C within 3 hours of delivery. A combined direct injection and LC-MS/MS assay (AbsolutIDQ p180 kit, Biocrates Life Sciences AG, Innsbruck, Austria) was used for the metabolomic analyses of the samples. Targeted metabolomic analysis showed a significant alteration between study groups in 29 metabolites from 3 distinct classes (Amino Acids, Acylcarnitines, and Glycerophospholipids). 9 of these metabolites were only significantly altered between neonates with Hypoxic ischaemic encephalopathy and matched controls, while 14 were significantly altered in both study groups. Multivariate Discriminant Analysis models developed showed clear multifactorial metabolite associations with both asphyxia and HIE. A logistic regression model using 5 metabolites clearly delineates severity of asphyxia and classifies HIE infants with AUC = 0.92. These data describe wide-spread disruption to not only energy pathways, but also nitrogen and lipid metabolism in both asphyxia and HIE.

**Conclusion:**

This study shows that a multi-platform targeted approach to metabolomic analyses using accurately phenotyped and meticulously biobanked samples provides insight into the pathogenesis of perinatal asphyxia. It highlights the potential for metabolomic technology to develop a diagnostic test for HIE.

## Introduction

Hypoxic ischaemic encephalopathy (HIE) occurs in 2 per 1000 deliveries [1], and causes 1 million neonatal deaths globally per year [2]. The hypoxic ischaemic (HI) insult is a complex injury, characterised by biphasic depletion in high energy phosphates. ATP levels initially decrease concurrently with the insult, recover following resuscitation, only to become depleted hours later, producing a secondary energy failure [3]. This secondary energy failure has been shown to be related to long-term outcome, and it is this step that current interventions, such as therapeutic hypothermia, attempt to attenuate [4], [5].

Therapeutic hypothermia improves outcome in neonates with moderate and severe HIE if initiated in the first 6 hours [5]. However the ability to recognise and diagnose those who would benefit from therapy is not always possible within this time period. Current standard methods to identify those at risk of HIE, are known to be unreliable [6], [7], and clinical grading systems such as the Sarnat score are most accurate beyond the time period for initiation of treatment [5], [8], [9].

Alternate methods of early assessment include neuro-imaging and neurophysiological monitoring, however early MRI is often impractical and can underestimate the degree of injury sustained [10]. Cotside neonatal electroencephalogram (EEG) is reliable in grading encephalopathy and an excellent predictor of long term outcome [11]. However a highly specialised skill set is required, which is rarely available to the neonatologist in the acute setting. Although amplitude integrated EEG (aEEG) is more widely used by clinicians, its interpretation is user dependent [12], and most neonatologists report they are not confident in their own interpretive ability [13]. This lack of a widely available objective measure has created the need for new markers of injury severity, to ensure all infants receive appropriate care.

The above findings have led to interest in blood based biomarkers for both the early grading of injury and the prediction of long-term outcome [14], [15], [16]. To-date most of this work has focused on targeted analysis of individual proteins [16], [17], [18], [19]. These proteins are chosen based upon our current understanding of the disease mechanism in HIE, but as mentioned above this is a complex disease for which the exact mechanism of which has yet to be fully elucidated. The injury is known to be multifactorial, involving energy depletion, the accumulation of intracellular Ca^++^ and excitatory amino acid release secondary to membrane depolarisation and failure of ATP dependent ion pumps, and the production of reactive oxygen species, culminating in mitochondrial membrane permeability and cell death [1], [20]. Therefore analysis of an individual protein in isolation over simplifies the injury that occurs. Such methods lack the adaptive potential of a systems-based approach, being unable to incorporate any dynamic interaction into a predictive model, and provide limited further insight into the disease mechanism.

Metabolomics is a systems biology strategy for exploring the low molecular weight biochemicals (metabolites) present in the metabolome of an organism [21]. It provides a description of the metabolic pathways activated, and their relation to one another, therefore affording a systems biology description of the disease mechanism leading to secondary energy failure, instead of isolated views of the process. Metabolomics details the final metabolic phenotype of the HI injury, reflecting the impact and interaction of the individual’s genomic, and transcriptomic input, as well as the HI injury sustained. In addition to further elucidating the disease process, a biochemical signature of HIE could be used to recognise injury severity. A number of animal metabolomic models of perinatal asphyxia have been recently published, highlighting the potential benefits of metabolomic analysis in human neonatal studies [22], [23], [24], [25].

To date no human studies of serum metabolomic analysis in neonatal HIE have been published. The aim of this study was to describe and quantify the metabolomic profile of umbilical cord blood samples in a carefully defined group of full term infants with perinatal asphyxia and HIE, using strict standard operating procedures for the collection, processing and analysis of samples.

## Materials and Methods

### Patient Selection

The study was conducted from May 2009 to June 2011 in a single maternity hospital with 9000 deliveries per annum. Ethical approval was obtained from the Clinical Research Ethics committee of the Cork Teaching Hospitals. Infants over 36 weeks gestation were eligible for inclusion if they had one or more of these previously described risk factors for asphyxia [26], [27], [28], [29]; an arterial cord pH <7.1, 5 minute Apgar score ≤6, or resuscitation at delivery required intubation. Parents of neonates meeting inclusion criteria were approached and written informed consent was obtained. Demographic and clinical details were prospectively recorded. Each infant had a HIE grade based on the modified Sarnat staging system assigned at 24 hours of age by a dedicated research fellow (BW) [8], [30]. The decision to initiate therapeutic hypothermia was at the clinicians’ discretion. When infants were cooled, total body hypothermia was used, cooling the infant to 33–34 degrees Celsius for 72 hours, as per the TOBY registry protocols (https://www.npeu.ox.ac.uk/tobyregister). The cases were divided into those with HIE, and those with biochemical or clinical risk of asphyxia without clinical encephalopathy (Asphyxia). Short term outcome was assessed using a standardised neurological assessment performed on day 3 and discharge [31].

All case infants had EEG monitoring during the first 24 hours of life. The EEG was commenced as soon as possible following delivery. Silver-silver chloride EEG electrodes were applied to the scalp at F3, F4, C3, C4, T3, T4, O2, O1 and Cz (according to the international 10 - 20 system of electrode placement, modified for neonates). The EEG was recorded using the NicOne video-EEG system (Carefusion, Madison, WI). The entire video - EEG was analyzed by an experienced neonatal electroencephalographer. The background EEG was graded as mild, moderate or severe according to a modification of a standardized HIE grading system [32], which we have previously described [11]. Electrographic seizures were identified, if there was a stereotyped repetitive discharge on one or more channels, with a clear evolution, that lasted for greater than 10 seconds.

A matched control population was recruited over the same period as part of an ongoing birth cohort study (The BASELINE Study www.baselinestudy.net). The controls and cases were matched for both infant and maternal demographic parameters including; gestational age, gender, birth weight and centile, method of delivery, maternal ethnicity, maternal age, and maternal body mass index (BMI). Ante-natal parental consent was obtained for all control infants enrolled. The control population had no clinical signs of asphyxia, or other medical issues at delivery. Clinically they were healthy, had normal examinations, and did not have EEG monitoring.

### Sample Collection and Storage

Umbilical cord blood was drawn on all case and control infants using identical standard operating procedures. 6 ml of umbilical cord blood was drawn from the cord, and placed in a plain serum tube within 20 minutes of delivery of the placenta. The Serum tube was allowed to clot for 30 minutes at 4°C. It was then centrifuged at 2400×g for 10 minutes at 4°C. The serum was pipetted into a second spin tube, and centrifuged for a further 10 minutes at 3400×g at 4°C. Following the second spin, the serum was aliquoted into lithium heparin microtubes and stored at −80°C until analysis. The time from birth to samples being placed within the −80°C freezer was always under 3 hours.

### Metabolomic Analysis

Sample preparation and metabolomic analyses were performed at The Metabolomics Innovation Centre (TMIC), University of Alberta, Canada. A targeted quantitative metabolomics approach using a combined Direct Flow Injection (DFI-) and liquid chromatography (LC-) MS/MS assay (AbsolutIDQ™ p180 kit) was used for the metabolomic analyses of the samples. The kit is a commercially available assay from Biocrates Life Sciences AG (Innsbruck, Austria). This kit assay, in combination with a 4000 QTrap (Applied Biosystems/MDS Sciex, Concord, Ontario, Canada) mass spectrometer, allowed simultaneous quantification of 148 metabolites (including 19 Acylcarnitines, 71 Phosphatidylcholines, 10 Lysophosphatidylcholines, 15 Sphingolipids, 21 Amino Acids, and 10 Biogenic Amines). A detailed list of all analysed metabolites is provided in Table S1. The method combines the derivatization and extraction of analytes with the selective mass-spectrometric detection using multiple reaction monitoring (MRM) pairs. Isotope-labelled internal standards are integrated into a kit plate filter for metabolite quantification.

**Figure 1:**
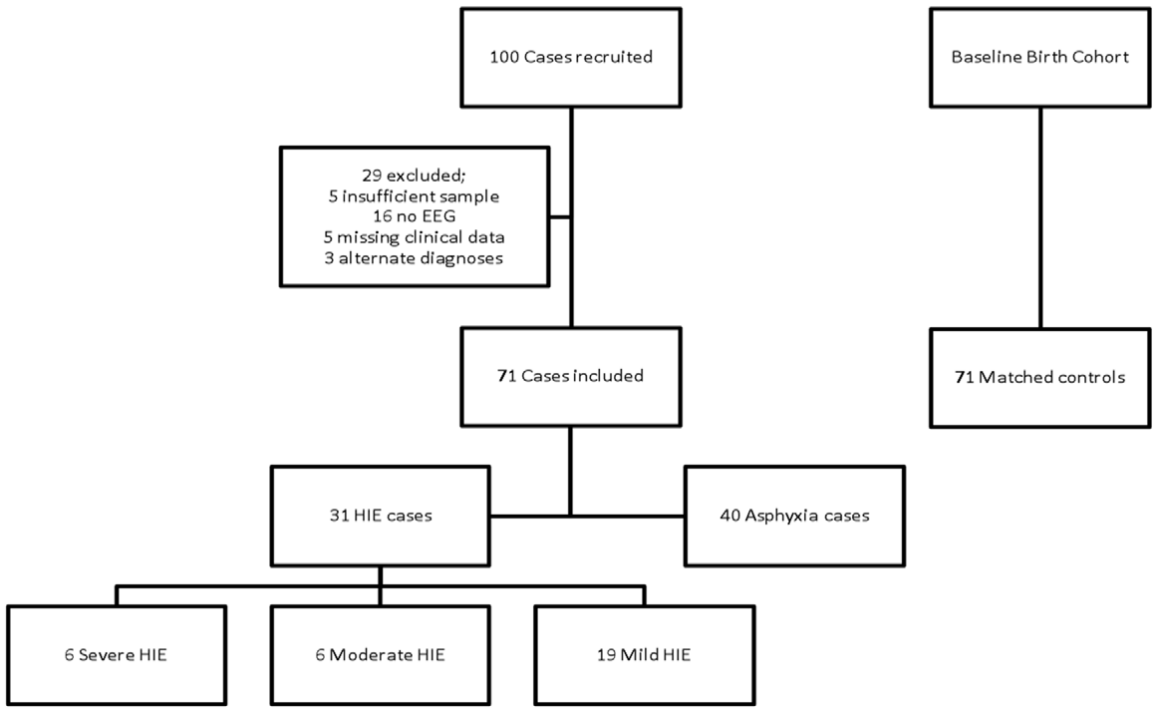
Flow diagram detailing enrolment of study infants.

The AbsoluteIDQ™ p180 kit contains a 96 deep-well plate with a filter plate attached with sealing tape, as well as reagents and solvents used to prepare the plate assay. The first 14 wells in each kit are used for standardization and quality control. A straightforward sample preparation step was used for the assay, as described in the kit’s user manual. Serum samples were left to thaw on ice and then vortexed and centrifuged at 13,000×g. A total of 10 µl of supernatant from each serum sample was loaded on a filter paper placed on top of the kit plate and dried in a stream of nitrogen. Subsequently, 20 µl of a 5% solution of phenyl-isothiocyanate was added for derivatization. After incubation, the filter spots were dried again using an evaporator. Extraction of the metabolites was then achieved by adding 300 µl methanol containing 5 mM ammonium acetate. The extracts were obtained by centrifugation into the lower 96-deep well plate, followed by a dilution step with 600 µl of the kit’s mass spectrometry running solvent. The extracts were analyzed using a 4000 QTrap (Applied Biosystems/MDS Sciex) mass spectrometer. The samples were delivered to the mass spectrometer by a LC method followed by a FIA method. MRM detection was used for quantification. MetIQ software, which is proprietary to Biocrates and included in the kit, was used to control the entire assay workflow. This included sample registration to automated calculation of metabolite concentrations to the export of data into other data analysis programs.

**Table 1:** Demographic Details of Study Population.

	HIE	Control HIE	*P*	Asphyxia	Control Asphyxia	*P*
	(n = 31)	(n = 31)		(n = 40)	(n = 40)	
**Gestational Age** (wks)	40.3 (1.1)	40.3 (0.9)	0.84	40.1 (1.3)	40.1 (1.1)	0.96
**Gender** (M/F)	20/11	21/10	0.79	27/13	27/13	1.0
**Birth Weight** (gm)	3543 (595)	3502 (463)	0.76	3713 (576)	3645 (508)	0.58
**Birth Weight Centile**	40 (20, 73)	46 (16, 82)	0.92	65 (34, 85)	65 (25, 84)	0.86
**Method of Delivery**			0.81			1.0
SVD	6 (20%)	8 (26%)		14 (35%)	14 (35%)	
Instrumental	15 (48%)	15 (48%)		19 (48%)	19 (48%)	
Emergency LSCS	10 (32%)	8 (26%)		6 (15%)	6 (15%)	
Elective LSCS				1 (2%)	1 (2%)	
**Sarnat Score**						
Severe/Moderate/Mild	6/6/19	…		…	…	
**Therapeutic hypothermia**	11	…		…	…	
**EEG Background**						
Severe/Moderate/Mild-Normal	8/3/20	…		0/0/40	…	
**Outcome at discharge**						
Severe/Mild/Normal	7/4/20	0/0/31	0.001	0/1/39	0/0/40	0.314
**1^st^ pH**	6.96 (0.18)	7.28 (0.18)	<0.001	7.04 (0.9)	7.22 (0.12)	<0.001
**1 minute Apgar score**	3 (1, 5)	9 (9, 9)	<0.001	6 (3, 7)	9 (9, 9)	<0.001
**5 minute Apgar score**	6 (3, 7)	10 (10, 10)	<0.001	8 (6, 9)	10 (9, 10)	<0.001
**Maternal Ethnicity**			0.08			0.56
Western European	26 (84%)	30 (96.7%)		38 (95%)	39 (97.5%)	
African	2 (6.4%)					
Indian	3 (9.6%)	1 (3.3%)				
Asian				2 (5%)	1 (2.5%)	
**Maternal Age** (yrs)	26.8 (5.0)	29.0 (4.9)	0.32	30.2 (6.3)	30.4 (4.9)	0.57
**Maternal BMI** kg/m^2^	25.2 (3.9)	24.0 (4.8)	0.35	26.1 (4.2)	24.2 (3.6)	0.06

Values are mean (SD), median (interquartile range), or n (%).

As an additional level of quality assurance, and to measure the precision and repeatability of the metabolite quantification (Table S1), serum from two of the control patients were repeatedly (and alternately) introduced into the analysis after every 9^th^ test sample. This resulted in 16 external quality control samples (8 from each patient). The remaining test samples were randomly ordered, stratified by outcome, such that no experimental bias was introduced by plate location and therefore MS injection order. All analytical procedures were performed by TIMC in isolation, blinded to both the identity of the external QCs and the study outcome groups.

**Figure 2:**
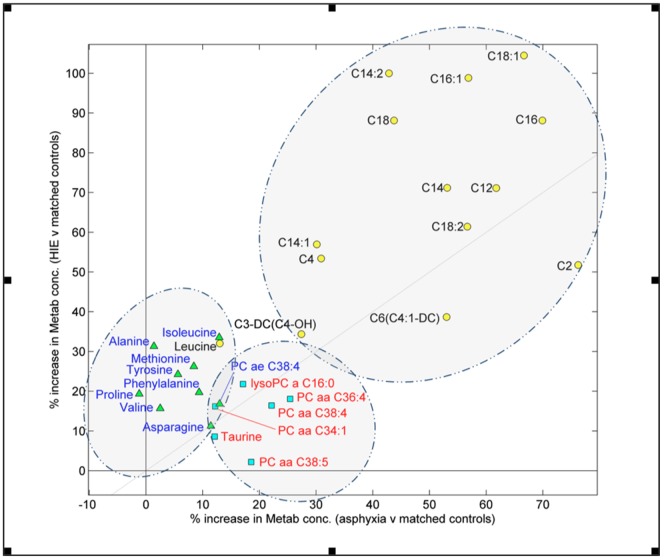
A comparison plot of percentage increase in metabolite concentration for metabolites that significantly differed in either the asphyxia vs. matched control comparison (squares), the HIE vs. matched controls (triangles), or both comparisons (circles).

**Figure 3:**
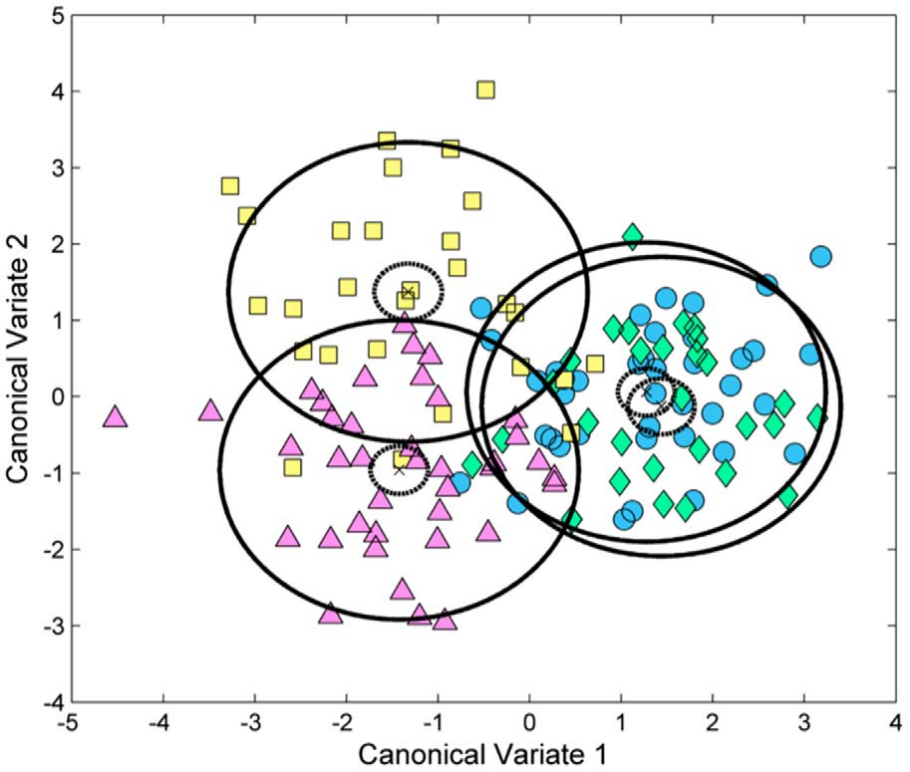
Canonical Variate Analysis for the combined data sets. Squares = asphyxia cases; Circles = matched asphyxia controls; Triangles = HIE cases; Diamonds = matched HIE controls. Solid circles = 95% confidence intervals for each group population; Dashed circles = 95% confidence intervals for the mean of each group.

**Table 2:** Metabolites Identified as significantly altered in Umbilical Cord Blood Samples of Cases compared to Matched Controls.

Metabolite		Asphyxia vs. Matched Control				HIE vs. Matched Control			
*Name*	*Class*	*p-value*	*q-value*	*median µM dif. in metab. conc.^§^ (95%CI)*	*% increase^‡^ (95%CI)*	*p-value*	*q-value*	*median µM dif. in metab. conc.^§^ (95%CI)*	*% increase^‡^ (95%CI)*
L-Acetylcarnitine [C2]	Acylcarnitines	**1.57E**−**05**	**0.001**	**2.85 (1.26,3.56)**	**76 (40,113)**	**8.16E**−**05**	**0.002**	**2.29 (1.50,2.66)**	**52 (37,84)**
Hydroxybutyrylcarnitine (Malonylcarnitine)[C3-DC (C4-OH)]	Acylcarnitines	**0.007**	**0.05**	**0.02 (0.00,0.04)**	**27 (**−**3,59)**	**0.003**	**0.02**	**0.03 (0.01,0.05)**	**34 (16,63)**
Butyrylcarnitine [C4]	Acylcarnitines	**6.28E**−**04**	**0.010**	**0.05 (0.03,0.06)**	**31 (25,56)**	**0.001**	**0.01**	**0.07 (0.02,0.10)**	**53 (13,89)**
Hexanoylcarnitine (Fumarylcarnitine) [C6 (C4∶1-DC)]	Acylcarnitines	**9.17E**−**04**	**0.01**	**0.04 (0.02,0.05)**	**53 (27,74)**	**4.34E**−**04**	**0.005**	**0.03 (0.02,0.06)**	**39 (20,84)**
Dodecanoylcarnitine [C12]	Acylcarnitines	**2.29E**−**04**	**0.004**	**0.03 (0.02,0.04)**	**62 (32,88)**	**8.96E**−**05**	**0.002**	**0.04 (0.02,0.08)**	**71 (38,141)**
Tetradecanoylcarnitine [C14]	Acylcarnitines	**2.42E**−**04**	**0.004**	**0.02 (0.01,0.03)**	**53 (27,82)**	**1.08E**−**04**	**0.002**	**0.03 (0.01,0.06)**	**71 (21,122)**
cis-5-Tetradecenoylcarnitine [C14∶1]	Acylcarnitines	**1.08E**−**04**	**0.004**	**0.03 (0.01,0.05)**	**30 (20,64)**	**1.72E**−**04**	**0.002**	**0.04 (0.02,0.06)**	**57 (26,109)**
3, 5-Tetradecadiencarnitine [C14∶2]	Acylcarnitines	**0.002**	**0.02**	**0.01 (0.00,0.01)**	**43 (17,57)**	**1.36E**−**04**	**0.002**	**0.01 (0.01,0.02)**	**100 (32,180)**
L-Palmitoylcarnitine [C16]	Acylcarnitines	**2.29E**−**04**	**0.004**	**0.06 (0.04,0.09)**	**70 (45,93)**	**5.05E**−**05**	**0.002**	**0.09 (0.05,0.14)**	**88 (47,127)**
Hexadecenoylcarnitine [C16∶1]	Acylcarnitines	**2.13E**−**05**	**0.001**	**0.02 (0.01,0.03)**	**57 (44,100)**	**2.79E**−**05**	**0.001**	**0.04 (0.01,0.05)**	**99 (47,154)**
Stearoylcarnitine [C18]	Acylcarnitines	**0.005**	**0.05**	**0.01 (0.00,0.01)**	**44 (24,65)**	**8.15E**−**05**	**0.002**	**0.02 (0.01,0.03)**	**88 (45,126)**
Oleoylcarnitine [C18∶1]	Acylcarnitines	**2.14E**−**05**	**0.001**	**0.04 (0.02,0.05)**	**67 (39,123)**	**2.79E**−**05**	**0.001**	**0.07 (0.03,0.10)**	**104 (47,140)**
Linoelaidyl carnitine [C18∶2]	Acylcarnitines	**2.17E**−**04**	**0.004**	**0.02 (0.01,0.02)**	**57 (33,87)**	**8.50E**−**06**	**0.001**	**0.02 (0.02,0.05)**	**61 (45,163)**
Alanine	Aminoacids	0.21	0.37	6.0 (−49.0,151.0)	1 (−10,34)	**0.001**	**0.01**	**140.0 (91.3,216.5)**	**31 (20,50)**
Asparagine	Aminoacids	0.05	0.20	5.4 (−0.8,10.5)	11 (−1,28)	**0.003**	**0.02**	**4.8 (1.8,15.9)**	**11 (3,34)**
Isoleucine	Aminoacids	0.03	0.14	8.6 (3.0,18.0)	13 (4,28)	**1.88E**−**04**	**0.002**	**21.1 (13.3,26.5)**	**34 (19,44)**
Leucine	Aminoacids	**0.009**	**0.07**	**17.0 (1.0,34.0)**	**13 (1,27)**	**6.17E**−**04**	**0.007**	**40.0 (28.0,57.0)**	**32 (13,44)**
Methionine	Aminoacids	0.14	0.30	2.1 (−0.7,4.4)	8 (−2,18)	**0.001**	**0.01**	**6.8 (3.5,10.2)**	**26 (11,37)**
Phenylalanine	Aminoacids	0.04	0.18	6.0 (0.5,8.7)	9 (1,12)	**0.001**	**0.010**	**16.7 (3.7,23.2)**	**20 (8,34)**
Proline	Aminoacids	0.91	0.96	−2.0 (−13.0,9.0)	−1 (−8,7)	**0.004**	**0.02**	**29.5 (11.6,54.5)**	**19 (8,39)**
Tyrosine	Aminoacids	0.06	0.22	3.8 (−1.4,17.9)	6 (−3,25)	**7.51E**−**04**	**0.007**	**16.8 (10.3,21.0)**	**24 (15,37)**
Valine	Aminoacids	0.21	0.37	5.0 (−12.0,38.0)	3 (−6,16)	**1.96E**−**04**	**0.002**	**31.0 (15.5,75.0)**	**16 (8,40)**
lysoPC a C16∶0	Glycerophospholipids	**0.002**	**0.02**	**8.7 (3.5,21.3)**	**17 (5,37)**	0.03	0.10	11.8 (4.2,18.6)	22 (−5,35)
PC aa C34∶1	Glycerophospholipids	**0.001**	**0.02**	**18.6 (0.0,43.0)**	**12 (0,39)**	0.12	0.19	19.5 (−1.0,38.0)	16 (−2,30)
PC aa C36∶4	Glycerophospholipids	**6.60E**−**04**	**0.01**	**33.0 (6.50,56.00)**	**25 (4,36)**	0.02	0.10	24.5 (2.0,59.0)	18 (−1,40)
PC aa C38∶4	Glycerophospholipids	**0.001**	**0.01**	**19.4 (9.4,31.2)**	**22 (12,35)**	0.01	0.07	15.1 (7.6,40.0)	16 (8,46)
PC aa C38∶5	Glycerophospholipids	**0.005**	**0.05**	**4.4 (2.4,8.7)**	**19 (9,31)**	0.27	0.32	0.5 (−1.6,8.5)	2 (−7,37)
PC ae C38∶4	Glycerophospholipids	0.09	0.25	1.0 (−0.5,2.4)	13 (−6,27)	**0.004**	**0.03**	**1.4 (0.5,2.5)**	**17 (5,33)**
Taurine	Biogenic Amines	**0.009**	**0.07**	**17.0 (7.0,45.0)**	**12 (4,28)**	0.16	0.22	14.0 (−10.0,36.0)	9 (−5,25)

### Statistical and Metabolomic Analysis

Statistical comparisons of clinical data between cases and controls were performed using Student’s t test, Mann–Whitney test, χ^2^ test or Fisher exact test, as appropriate.

Metabolites measured with more than 20% missing data were dropped from subsequent statistical analysis. Relative Standard Deviation (RSD) for the two sets of QC samples was calculated for each metabolite. Data was then log transformed. Analysis of covariance was performed comparing each metabolite to each clinical variable in turn, in order to assess potential bias in the study design.

**Figure 4:**
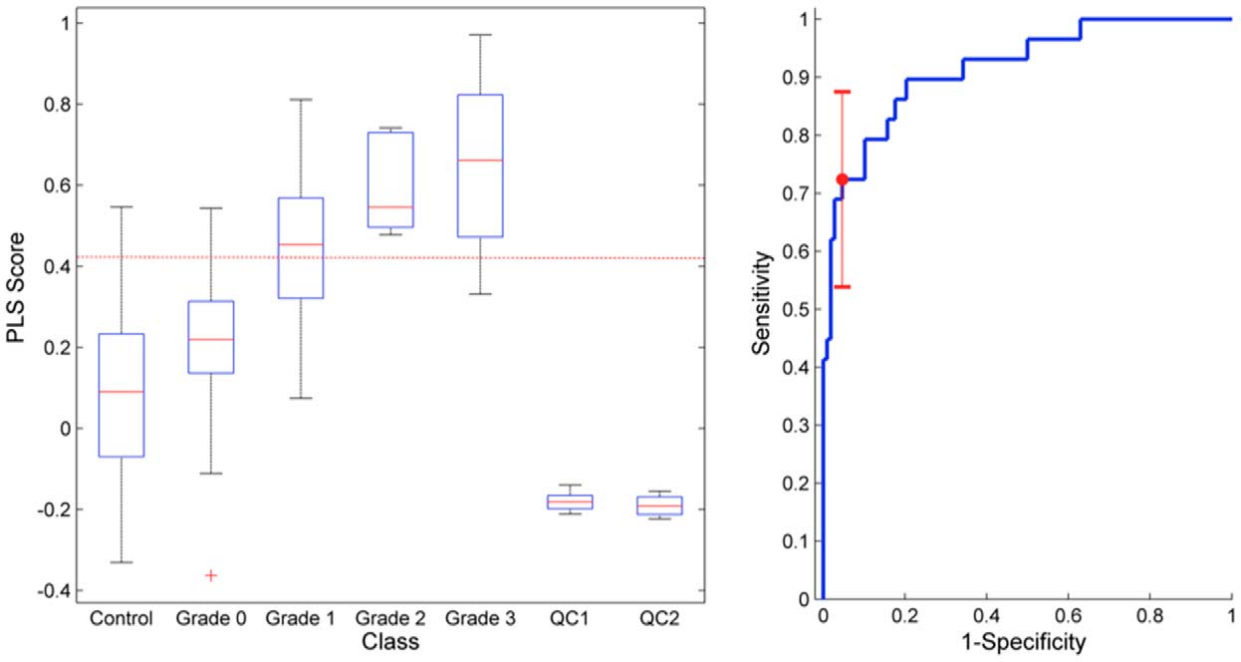
The predictive scores for a PLS-DA model built to discriminate between HIE versus all other outcomes (asphyxia and both the control groups) using the complete data set. The PLS score box plot is grouped by Sarnat score. Here a Sarnat score of zero is equivalent to the “asphyxia” classification, and Sarnat grade of 1, 2 and 3 represent the 3 levels of increasing HIE severity. The model was optimally built using 2 latent factors. The model had an R^2^ = 0.32, Q^2^ = 0.22, and an AUC of 0.92 (95% CI: 0.84–0.97). For a fixed specificity of 0.95 the corresponding sensitivity for predicting HIE (at any level) is 0.75 (95% CI: 0.55–0.88), the corresponding decision boundary is indicated by a dashed line in the boxplot. Note: the Quality Control samples (repeated injection of serum from two control patients: QC1 & QC2) are projected through the PLS-DA model and the subsequent predictions give an estimation of model precision.

**Figure 5:**
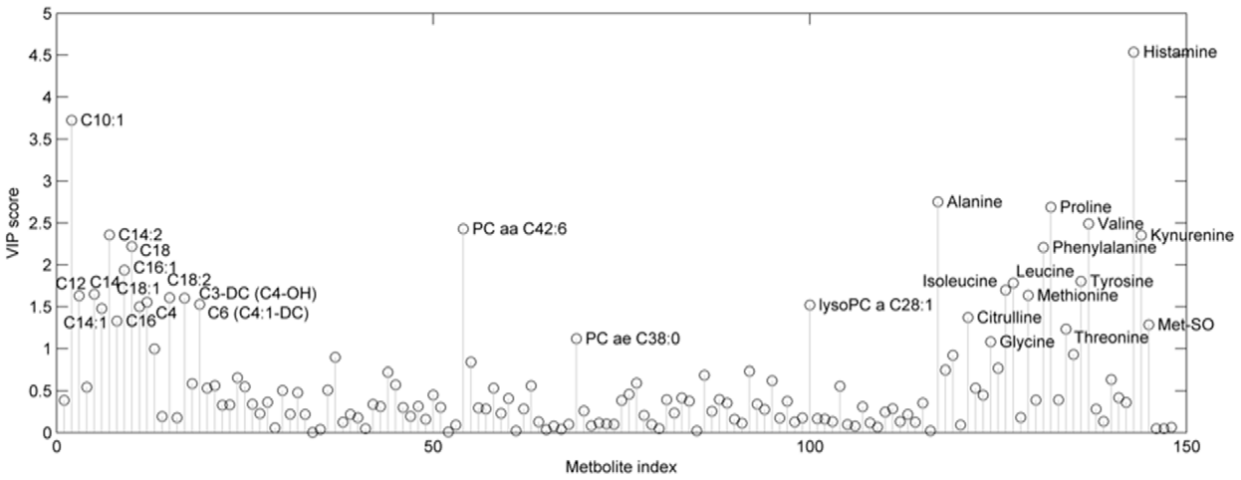
Variable importance plot for the HIE versus ‘all other outcomes’ PLD-DA model. A VIP score >1 indicates an important contribution to the model.

For each metabolite reproducibly detected, the null hypothesis that the means of matched case and control sample populations were equal was tested using paired Wilcoxon signed rank test. Correction for multiple comparisons was performed using the method described by Benjamini and Hochberg [33]. Both p-values and corrected q-values are reported. The median absolute difference (µM), and median percentage increase, in paired case versus control samples was also reported with 95% confidence intervals.

In order to compare the univariate results from the two arms of this study (*HIE vs. control* and *asphyxia vs. control*) a bi-plot of median percentage increase for those metabolites significant in either comparison was constructed. In addition, for the combined sample populations, Canonical Variate Analysis (CVA) [34] was performed on this set of differentially changing metabolites in order to visualise the associated multifactorial and correlated discrimination between all 4 groups.

**Figure 6:**
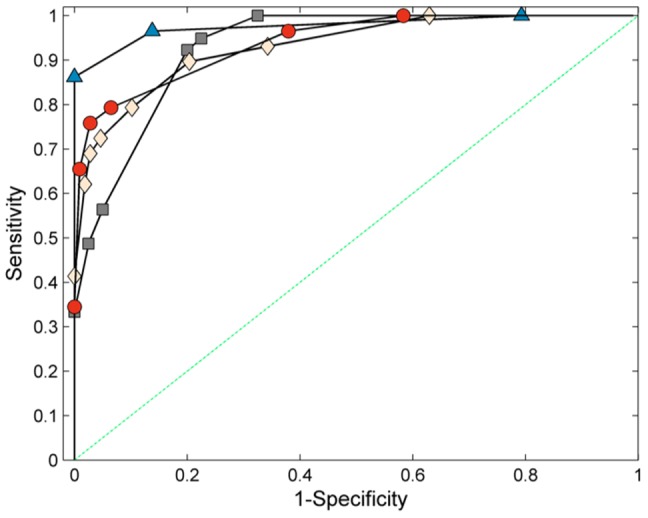
A ROC comparison of all models produced in this study. Triangle = PLS-DA: HIE versus matched controls, AUC = 0.96 (95% CI = 0.83–1.00); Square = PLS-DA: Asphyxia versus matched controls, AUC = 0.91 (0.83–0.96); Diamond = PLS-DA: HIE versus ‘all other outcomes’ (all metabolites), AUC = 0.91 (0.83–0.96); Circle = Logistic Regression: HIE versus ‘all other outcomes’ (5 metabolites), AUC = 0.92 (0.84–0.97). For clarity the convex-hull ROC curve approximations are shown. All AUC calculations were made on the actual predicted values.

**Table 3:** Logistic regression model for HIE versus ‘all other outcomes’: AUC of 0.92 (95% CI: 0.83–0.96) A specificity of 0.95 produced a corresponding sensitivity of 0.72 (95% CI: 0.54–0.88).

Metabolite	Class	β	Std (β)	z-score	P>|z|	β - 95% Conf.	
Decenoyl-L-carnitine	Acylcarnitines	−19.52	−0.77	−3.8	<0.001	−29.59	−9.45
3, 5-Tetradecadiencarnitine	Acylcarnitines	12.12	0.78	2.78	0.005	3.58	20.67
PC ae C38∶0	Glycerophospholipids	−9.83	0.82	−2.71	0.007	−16.95	−2.71
Phenylalanine	Aminoacids	17.48	−0.76	3.19	0.001	6.74	28.22
Proline	Aminoacids	10.22	0.84	4.42	<0.001	5.69	14.75
constant offset β_0_	–	−92.32	–	−3.16	0.002	−149.66	−34.98

Standardised parameter values (Std (β)) indicate an equal contribution from all constituent metabolites. Statistical analysis (z-score) of the β values indicated that all the constituent metabolites significantly contributed to the model.

Multivariate profile-wide predictive models were constructed using Partial Least Squares Discriminant Analysis (PLS-DA) [35], [36]. For each model, all of the reproducible metabolites for a given comparison were included. For each metabolite, data were mean centred, and scaled to unit variance [37]. Missing values were imputed using the k-nearest neighbour method [38]. The number of latent variables in each model was selected using stratified 10-fold cross validation, and associated R^2^ and Q^2^ statistics calculated. Here, R^2^, the squared correlation coefficient between the dependent variable and the PLS-DA prediction, measures “goodness-of-fit” (a value between zero and one, where one is a perfect correlation). Q^2^ provides a measure of “goodness-of-prediction” and is the averaged correlation coefficient between the dependent variable and the PLS-DA predictions for the 10-fold out data sets generated during the cross-validation process. Further validation was performed to check the robustness of the final PLS-DA model by comparing it’s R^2^ value to a reference distribution of all possible models using permutation testing (N = 1000) following the standard protocol for metabolomic studies [39], [40]. Here a reference R^2^ distribution is obtained by calculating all possible PLS-DA models under random reassignment of the case/control labels for each measured metabolic profile. If the correctly labelled model’s R^2^ value is close to the centre of the reference distribution then the model performs no better than a randomly assigned model and is therefore invalid. For all PLS-DA models described here the associated reference distribution plots are provided, from which an estimate of the probability of the candidate model randomly occurring can be estimated. In addition, a receiver operating characteristic (ROC) curve was determined for each model (including bootstrap 95% confidence intervals for specified model specificity) such that an accurate assessment of discriminatory ability could be made [41]. For each PLS-DA model, variable importance values in the projection (VIP) are computed according to Chong and Jun [42].

Finally, Backward Stepwise Logistic Regression (BSLR) [43] was performed in order to determine the most parsimonious model for discriminating between HIE samples and the combined asphyxia plus normal samples. Covariate corrected ROC analysis was performed to assess the potential confounding influence of pertinent clinical variables [41].

All of the statistical analyses were carried out using the Matlab scripting language (http://www.mathworks.com/), with the exception of the logistic regression which was performed using STATA 12.0 (http://www.stata.com/).

## Results

### Study Population

100 neonates meeting inclusion criteria were recruited to this study. 29 of these were subsequently excluded (5 had insufficient cord samples; 16 had no EEG; 5 had missing clinical details; 3 had an alternate diagnosis [1 perinatal infarction, 1 neuromuscular disease, 1 sepsis]) leaving a study population of 71, 31 with clinical HIE, and 40 with asphyxia but not encephalopathic (Figure 1). Among the 31 infants with clinical HIE, 6 had a severe Sarnat score, 6 moderate, and 19 mild. In addition, 70 of the 71 cases had early multichannel-EEG, the 1 infant that did not, was a severe case of HIE who did not survive beyond the delivery room. The grade of encephalopathy assigned by early multichannel–EEG strongly correlated and supported the clinical grade (Spearman’s correlation coefficient = 0.779, p<0.001). 6 cases with encephalopathy developed electro-clinical seizures, and 5 died. 11 infants with HIE received therapeutic hypothermia, 1 infant with severe HIE did not survive to the neonatal unit. 71 healthy matched control infants were recruited.

There was a strong correlation between Sarnat score and short-term outcome in infants with HIE (*r* = 0.884, p<0.001; *r* = 0.807, p<0.001; for Amiel-Tison on day 3 and at discharge respectively). Specifically in all those with severe HIE (n = 6), assessment of short-term outcome demonstrated a severely abnormal Amiel-Tison (or death) on day 3 and discharge. 4 infants with moderate HIE had a severely and 1 a mildly abnormal Amiel-Tison on day 3, which by discharge improved to severely abnormal in 1, mildly abnormal in 1, and normal in 4. In those with mild HIE the Amiel-Tison on day 3 was mildly abnormal in 5 and normal in 14, and by discharge mildly abnormal in 3 and normal in 16. Of the infants with Asphyxia, 1 had a mildly abnormal Amiel-Tison at discharge (reduced thumb abduction), all others were normal, and similarly all of the controls had a normal newborn examination. The demographic and clinical details are given in table 1. There were no clinical or laboratory evidence of maternal or neonatal infection in any of the infants studied.

### Umbilical Cord Serum Analysis

148 metabolites were reproducibly measured in serum obtained from the umbilical cord blood from each of the 142 infants studied. Analysis of covariance showed that there was no significant association between any of the measured metabolites and the potential confounding clinical variables in this study (gestational age, gender, birth weight and centile, method of delivery, maternal age and ethnicity, and maternal BMI). Assessment of the Quality Control measurements revealed an average Relative Standard Deviation 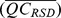 of 5%, an average biological signal-to-noise ratio of 23 dB. See Table S1 for details. Only one metabolite had more than 20% missing values.

Using a critical p-value of <0.01, 29 metabolites were found to be significantly different between either asphyxia vs. matched controls or HIE vs. matched controls (Table 2). Of the 29 significant metabolites, 14 differed significantly in both comparisons, 9 differed significantly in only the HIE vs. matched controls comparison, and 6 differed significantly in only the asphyxia vs. matched controls comparison. Figure 2 describes the percentage increase in metabolite concentrations for only the significant metabolites in either arm of the study. The plot clearly shows three clusters of metabolite classes. Glycerophospholipids were significantly altered in the Asphyxia versus Control comparison, Amino Acids significantly altered in the HIE versus Control comparison and Acylcarnitines were significantly altered in both comparisons. It is important to note that whilst the Acylcarnitines cluster was significant in both comparisons these metabolites are more significantly increased for the HIE infants compared to the asphyxia infants. Canonical Variate Analysis of the combined data sets (all 142 infants) confirms the univariate results (Figure 3). The first canonical variate (CV1) describes a significant multivariate mean difference between cases and controls for both HIE and asphyxia outcomes. The second canonical variate (CV2) describes an orthogonal and significant multivariate mean difference between the HIE and asphyxia group but not the control groups. Note that there was no significant difference between the two control groups in either CV1 or CV2.

Cross-validated Partial Least Square Discriminant Analysis (PLS-DA) models for HIE versus matched controls, and asphyxia versus matched controls, were built using two latent factors (Figure S1 and Figure S2). The HIE versus matched controls model had an R^2^ = 0.59, Q^2^ = 0.43, and an AUC of 0.96 (95% CI: 0.83–1.00). The asphyxia versus matched controls model had an R^2^ = 0.43, Q^2^ = 0.23, and an AUC of 0.91 (95% CI: 0.83–0.96). Permutation testing showed that the probability of any of these models randomly occurring was less than 0.001 (Figure S1 and Figure S2). Variable importance plots for both these models showed similar discriminatory metabolite profiles localised to the Acylcarnitine, Amino Acid, Glycerophospholipid metabolite classes (Figure S3).

The data from all infants was then combined, and a PLS-DA model for HIE versus all other outcomes (asphyxia and both the control groups) constructed. Once again this model was built using two latent factors. The HIE model had an R^2^ = 0.32, Q^2^ = 0.22, and an AUC of 0.92 (95% CI: 0.84–0.97) (Figure 4). Permutation testing showed that the probability of this models randomly occurring was less than 0.001 (Figure S4). Figure 4 describes the PLS-DA model predictions. The box-plot is grouped by modified Sarnat score. Here a Sarnat score of zero is equivalent to the “asphyxia” classification, and Sarnat score of 1, 2 and 3 represent the 3 levels of HIE severity. For a fixed specificity of 0.95 the corresponding sensitivity for predicting HIE (at any level) is 0.75 (95% CI: 0.55–0.88). The associated variable importance plot shows a discriminatory metabolite profile consisting of mainly Acylcarnitines and Amino Acids (Figure 5).

Using the combined infant populations, backward stepwise logistic regression was performed using the metabolite subset described in Figure 5 (VIP>1) as a starting point. It was possible to find a parsimonious model to discriminate HIE from all other outcomes using only 5 metabolites (Table 3). All 5 metabolites significantly contributed to the logistic model (p<0.01), thus minimising the probability of the model fitting to random multivaraite associations in the data. This model produced an AUC of 0.92 (95% CI: 0.83–0.96) and for a fixed specificity of 0.95 produced a corresponding sensitivity of 0.72 (95% CI: 0.54–0.88). The associated ROC curve was not significantly different to that of the associated PLS-DA model. Figure 6 compares the ROC curves for all the described PLS-DA models together with the 5-metabolite logistic regression model.

Finally, covariate corrected ROC analysis was performed to assess the potential confounding influence of the clinical variables described in Table 1 (Method of Delivery; Gender; Birth Weight; Gestational Age; Maternal Age; Maternal BMI) on the uncorrected logistic regression predictions described above. A linear covariate adjusted model was used (STATA:rocreg), together with nonparametric bootstrap AUC estimates. The adjusted ROC curve produced an AUC of 0.93 (95% CI: 0.81–0.99), which was a marginal, but non-significant, improvement in prediction when compared to the uncorrected model. Statistical analysis of the parameter values in the linear adjustment model confirmed that none of the potential confounding factors had any significant influence on the uncorrected ROC curve (Table S2).

## Discussion

To accommodate the complex multi-factorial nature of neonatal hypoxic-ischaemic injury, this study used metabolomic techniques to describes the biochemical derangement present in the cord blood of a carefully defined cohort, with clinical and EEG evidence of HIE.

Targeted metabolomic analysis showed a significant increase in 29 of the 148 measured metabolites in the umbilical cord blood of infants with either asphyxia or HIE compared to matched healthy controls. Three distinct metabolite classes were disrupted: Amino Acids, Acylcarnitines, and Phosphatidylcholines (Table 2 & Figure 2). Of these, a group of 8 amino acids were significantly increased in neonates with HIE relative to matched controls, but not in the asphyxia group. While a group of 13 acylcarnitines were significantly increased in both study groups; however, for the acylcarnitines the increase was more pronounced in the HIE population. Canonical Variate Analysis (Figure 3) suggested that the major disruption in the metabolomic profile is due to asphyxia severity (CV1), dominated by the acylcarnitines profile; however there is also an additional degree of orthogonal discrimination between HIE and asphyxia (CV2).

Given the sporadic nature and lack of antenatal warning for perinatal asphyxia, it can prove difficult to recruit and collect suitable samples in these infants. This has lead to multiple studies of neonatal asphyxia including a heterogeneous population of mixed gestational age [44], [45], and broad inclusion parameters [46], [47]. Recognising the complex nature and multiple potential aetiologies of this disease, we attempted to remove as many confounding variables as possible limiting our analyses to term infants, with the injury carefully defined using both modified Sarnat score and EEG. The modified Sarnat score was the primary means used to categorise grade of injury [8], [30], as this reflects standard clinical practice. The EEG was used to validate these findings as it is also a robust predictor of outcome, and has the advantage of being able to be assessed blindly [48], [49], [50]. Given the strong correlation (*r* = 0.779, p<0.001) between the two grading systems, and similarly strong correlation between clinical grade and short-term outcome, we were confident in the accuracy of the grade assigned for analysis.

The current study is limited by the lack of a separate validation cohort. This is due to the difficulty in recruitment and collection of cord blood samples in a timely fashion as discussed above. Despite this, there is strong supporting evidence for these findings from a recent animal model [25]. Solberg et al. drew plasma samples at the start and end of hypoxia in a piglet model, and biobanked the samples at −70°C pending analysis. The same 148 metabolites as described in the current study were analysed by Solberg et al. using the Absolut*IDQ* kit p150 (Biocrates Life Sciences AG, Innsbruck, Austria), plus a further 65 metabolites, including additional amino acids, biogenic amines, bile acids, oysterols, and organic acids were analysed using alternate techniques. Of the 148 metabolites analysed in both cohorts, 18 were significantly altered in the two, all from the acylcarnitine and amino acid metabolite classes. In all cases of agreement the direction of alteration was the same in both the animal model and the current study [51]. Given that Solberg et al. used similar methods for biobanking, identical methods of analysis, and that metabolites are largely species-independent, their findings are strongly supportive of the metabolomic profile described here. However it must be noted that in the animal model although all cases had perinatal asphyxia, the severity of brain injury was not quantified. As such the authors could only discuss their results in relation to asphyxia and not HIE.

Under aerobic conditions, energy production is a complex process with differing metabolite classes being catabolised through multiple interacting pathways. For fatty acids catabolism, they are transported across the inner mitochondrial membrane by carnitine, in the form of an acylcarnitine [52]. They are then activated by the addition of CoA and enter the β-oxidation pathway, producing FADH_2_, NADH, and Acetyl-CoA, while the liberated carnitine is recycled for further transport. The reducing equivalents proceed to enter the electron transport chain, with a net production of ATP, while the Acetyl-CoA enters the Krebs cycle, with further energy production. Amino acids are used in energy production by conversion to glucose, ketone bodies, or frequently key intermediates in the Krebs cycle (e.g. Isoleucine can produce either Acetyl-CoA, through condensation, or Succinyl-CoA through transamination) [53], [54].

In the event of Hypoxia-ischemia, there is a switch to anaerobic metabolism, and a breakdown in these normal processes [55]. An animal model of Hypoxia-ischemia demonstrated that the reduced rate of β oxidation, likely due to an alteration in the NADH/NAD and FADH_2_/FAD ratios, becomes the rate limiting step for fatty acid metabolism [56]. In animals this results in an increase in matrix long-chain acyl-CoA and their upstream pre-cursors, the long-chain acylcarnitines. This increase in levels of acyl-CoA, and long chain acylcarnitines cause further disruption to oxidative metabolism, and can be directly toxic. Similarly the disruption of the Krebs cycle results in an increase in the cycle’s intermediaries and pyruvate breakdown products [25], [27], [57], [58]. This could result in an accumulation of their precursor amino acids, as described in this study. An additional explanation for the increase in the branched chain amino acids and alanine, found in this study, is that they are known to act as alternate energy sources, for the perinatal brain and muscles [59], potentially leading to their mobilisation under conditions of reduced energy production.

Studies of carnitine, and acylcarnitine profiles, in perinatal asphyxia using post-natal blood samples (collected from Day 1–7) have yielded conflicting results [60], [61], [62]. This is unsurprising as post-natal samples may be altered secondary to resuscitation [25], or the infant’s method of feeding, making their analysis unreliable. The only other study of human cord blood samples was conducted by Meyburg et al. [63]. The authors profiled healthy term neonates (n = 70), and found an inverse relationship between several short and long chain acylcarnitines, and both pH and 5 minute Apgar score. Although Meyburg et al. was looking at healthy neonates, the inverse relationship described is consistent with our findings.

In the current study, acylcarnitines were raised in infants with both asphyxia and HIE relative to controls, while amino acids were predominantly raised in those with HIE. Wainwright et al., hypothesised that high acyl-CoA levels may be “the earliest and a cardinal irreversible event in ischemia”, preceding the formation of oxygen free radicals and nitric oxide [64]. This is a potential explanation for the acylcarnitine rise in both case groups found in this study, implying an earlier alteration in the acylcarnitine profile following an asphyxia event, than alterations of amino acids. Wainwright was particularly interested in this possibility, as their animal model found that carnitine supplementation prior to the hypoxic event could attenuate the injury sustained in their models [64], [65].

In addition we demonstrate the potential for the metabolic signature present in the umbilical cord blood to distinguish injury severity, and therefore the possibility of directing the need for treatment. The multivariate discriminant analysis models (PLS-DA) showed clear multifactorial metabolite associations with both asphyxia and HIE (Figure 5). The PLS-DA model discriminating HIE from all other outcomes (Figure 4) indicated that the biomarker signature for HIE has the potential to predict severity of insult rather than a yes/no binary outcome. The more severe the modified Sarnat score the more accurate the classification. Using robust data mining and modelling techniques, we have shown that the metabolite profile in the cord blood at birth, representing the latent systems-wide interaction in the metabolome, is sufficient to produce a robust predictive model for presence of encephalopathy at 24 hours of age with an AUC of 0.93.

Perinatal asphyxia is a complex disease with a broad spectrum of injury, dependent on factors such as specific aetiology, duration of insult, time after injury, and intervention provided. The classical use of single markers to predict disease does not allow the adaptability required in such a multi-faceted disease entity, rather the use of a mutli-factorial model is required to appropriately define the injury. For this reason in the current study, single metabolites were not highly significant, while the multi-metabolite HIE model was highly predictive, and able to distinguish between all groups. While this PLS-DA model has not been validated in an independent human cohort, there is much similarity to a previously described animal model [25].

In summary, we have described the profile of the human metabolome in neonatal asphyxia for the first time, identifying an alteration in 29 metabolites from the glycerophospholipid, amino acid and acylcarnitine classes. Furthermore we have demonstrated the potential of this metabolic signature at birth, to predict on-going encephalopathy at 24 hours. With further validation these metabolites may offer the potential to improve prediction of injury severity at the time of birth.

## Supporting Information

Figure S1 A 10-fold cross-validated PLS-DA model built to discriminate between HIE versus matched controls using all 148 measured metabolites. (a) PLS-DA predictive scores (circles = HIE; Squares = Controls;+ =  QC1; × = QC2). The associated ROC curve had an AUC of 0.96 (95% CI: 0.83–1.00). (b) A plot of the R2 and Q2 values for a range of latent factors. The optimal number of latent factors to avoid over-fitting was determined to be equal to 2. The optimal model had an R2 = 0.59 and Q2 = 0.43 (c) A non-parametric test comparing the ‘candidate’ model (red line) and the randomly permuted H0 distribution (blue histogram) showed that the probability of a model of this quality randomly occurring was less than 0.001.
(TIFF)

Figure S2 A 10-fold cross-validated PLS-DA model built to discriminate between asphyxia versus matched controls using all 148 measured metabolites. (a) PLS-DA predictive scores (circles = asphyxia; Squares = Controls; + =  QC1; × = QC2). The associated ROC curve had an AUC of 0.91 (95% CI: 0.83–0.96). (b) A plot of the R2 and Q2 values for a range of latent factors. The optimal number of latent factors to avoid over-fitting was determined to be equal to 2. The optimal model had an R2 = 0.43 and Q2 = 0.23 (c) A non-parametric test comparing the ‘candidate’ model (red line) and the randomly permuted H0 distribution (blue histogram) showed that the probability of a model of this quality randomly occurring was less than 0.001.
(TIFF)

Figure S3 Variable importance plots for (a) the HIE versus matched controls PLD-DA model and (b) the asphyxia versus matched controls PLD-DA model. A VIP score >1 indicates an important contribution to the model.
(TIFF)

Figure S4 A cross-validated PLS-DA model to discriminate between HIE versus all other outcomes (asphyxia and both the control groups) using all 148 measured metabolites (a) The optimal number of latent factors to avoid over-fitting was determined to be 2, with an R2 = 0.32 and Q2 = 0.22. Model scores are shown in figure 3. (b) A non-parametric test comparing the ‘candidate’ model (red line) and the randomly permuted H0 distribution (blue histogram) showed that the probability of a model of this quality randomly occurring was less than 0.001.
(TIFF)

Table S1 List of quantified metabolites. Included for each metabolite: The recommended limit of detection for the specified platform; percentage of missing values (i.e. metabolite concentration below the measurable limit); mean serum concentration across the whole sample population; The Relative Standard Deviation (RSD) for the two repeat-injection Quality Control patients (8 reps per patient evenly dispersed across the experimental run) – an RSD of <20% is considered acceptable; The Biological Signal to Noise ratio (S/N) in decibels (dB) – this gives an indication of biological information content, calculated using the following equation: 20 log (RMSsample/RMSQC), where RMS = Root Mean Squared amplitude of the mean centred data. A S/N >15 dB indicates excellent information content.
(DOC)

Table S2 Table of the parameter estimates generated by the covariate adjusted ROC analysis (using non-parametric bootstrap AUC estimation and a linear correction model). The associated corrected area under the ROC curve (AUC) was 0.93 (95% CI: 0.81–0.99); this was not signifiacntly different to the uncorrected ROC curve. T-tests estimating the contribution of each clinical variable to the linear correction model also indicate that each clinical variable had no significant `influence on the corrected ROC curve. Thus we conclude that these potential confounders had neither a significant positive nor negative impact on the metabolite biomarker signature.
(DOC)
