# Supplementary figures and images for: The Metabolomic Profile of Umbilical Cord Blood in Neonatal Hypoxic Ischaemic Encephalopathy
Source: PLoS One. 2012 Dec 5;7(12):e50520. doi: 10.1371/journal.pone.0050520 (PMC3515614; doi:10.1371/journal.pone.0050520)

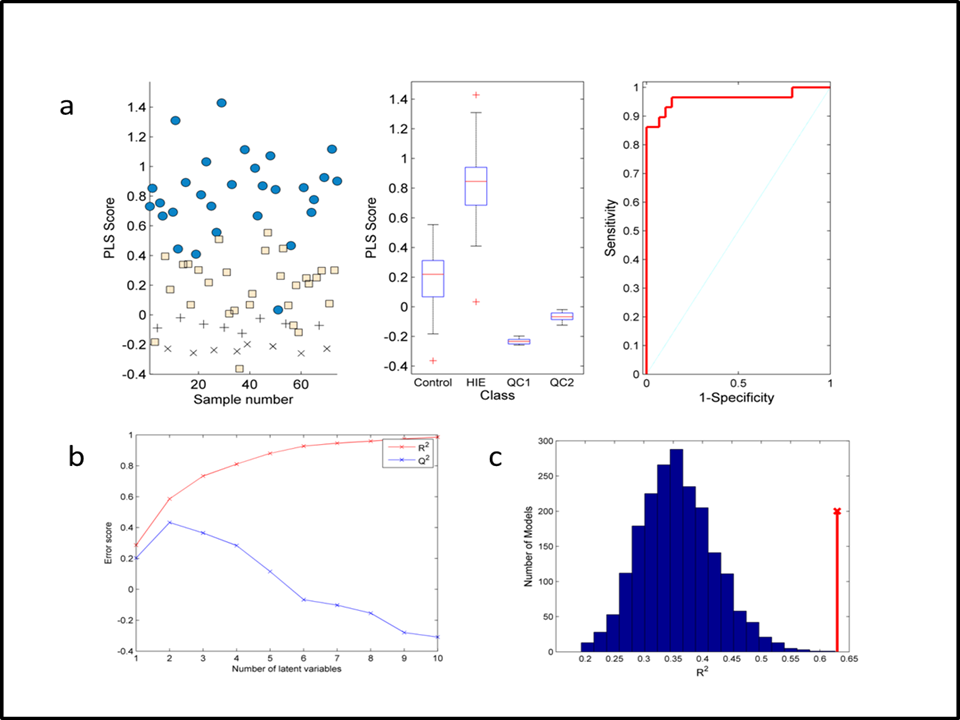

Supplement: Figure S1 — A 10-fold cross-validated PLS-DA model built to discriminate between HIE versus matched controls using all 148 measured metabolites. (a) PLS-DA predictive scores (circles = HIE; Squares = Controls;+ = QC1; × = QC2). The associated ROC curve had an AUC of 0.96 (95% CI: 0.83–1.00). (b) A plot of the R2 and Q2 values for a range of latent factors. The optimal number of latent factors to avoid over-fitting was determined to be equal to 2. The optimal model had an R2 = 0.59 and Q2 = 0.43 (c) A non-parametric test comparing the ‘candidate’ model (red line) and the randomly permuted H0 distribution (blue histogram) showed that the probability of a model of this quality randomly occurring was less than 0.001. (TIFF) [file pone.0050520.s001.tiff]

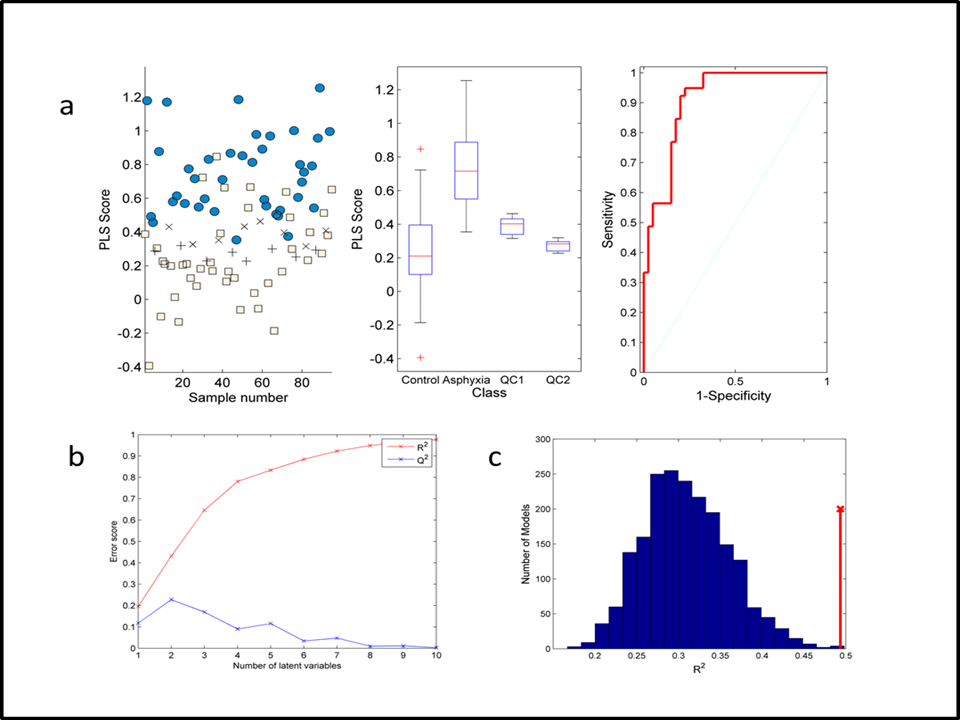

Supplement: Figure S2 — A 10-fold cross-validated PLS-DA model built to discriminate between asphyxia versus matched controls using all 148 measured metabolites. (a) PLS-DA predictive scores (circles = asphyxia; Squares = Controls; + = QC1; × = QC2). The associated ROC curve had an AUC of 0.91 (95% CI: 0.83–0.96). (b) A plot of the R2 and Q2 values for a range of latent factors. The optimal number of latent factors to avoid over-fitting was determined to be equal to 2. The optimal model had an R2 = 0.43 and Q2 = 0.23 (c) A non-parametric test comparing the ‘candidate’ model (red line) and the randomly permuted H0 distribution (blue histogram) showed that the probability of a model of this quality randomly occurring was less than 0.001. (TIFF) [file pone.0050520.s002.tiff]

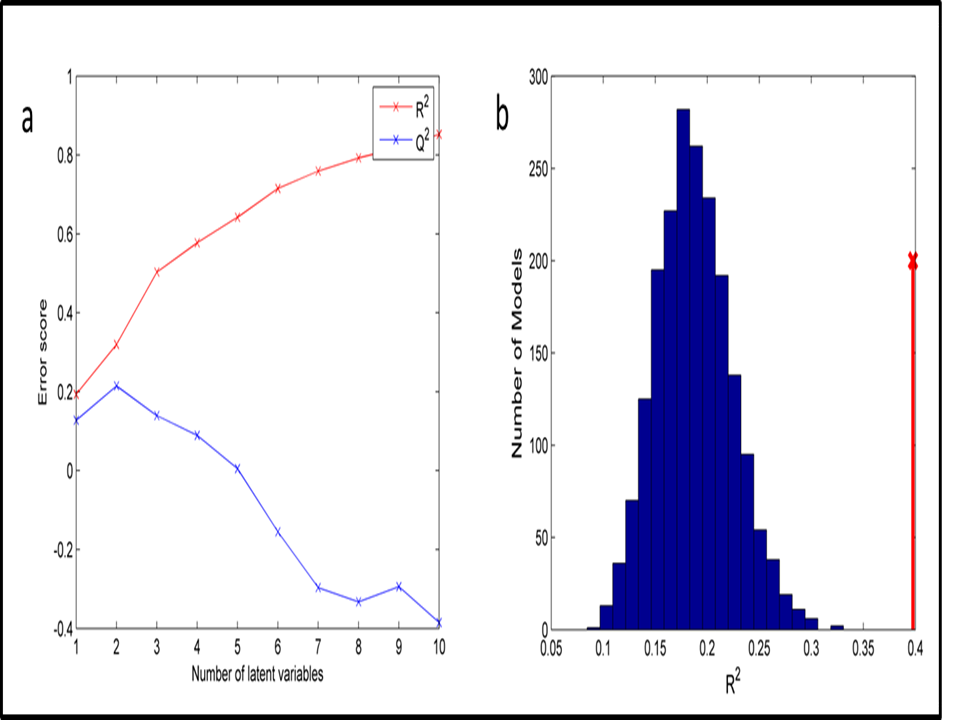

Supplement: Figure S4 — A cross-validated PLS-DA model to discriminate between HIE versus all other outcomes (asphyxia and both the control groups) using all 148 measured metabolites (a) The optimal number of latent factors to avoid over-fitting was determined to be 2, with an R2 = 0.32 and Q2 = 0.22. Model scores are shown in figure 3. (b) A non-parametric test comparing the ‘candidate’ model (red line) and the randomly permuted H0 distribution (blue histogram) showed that the probability of a model of this quality randomly occurring was less than 0.001. (TIFF) [file pone.0050520.s004.tiff]
