# Supplementary material for: The Metabolomic Profile of Umbilical Cord Blood in Neonatal Hypoxic Ischaemic Encephalopathy
Source: PLoS One. 2012 Dec 5;7(12):e50520. doi: 10.1371/journal.pone.0050520 (PMC3515614; doi:10.1371/journal.pone.0050520)
Supplement: Table S1 — List of quantified metabolites. Included for each metabolite: The recommended limit of detection for the specified platform; percentage of missing values (i.e. metabolite concentration below the measurable limit); mean serum concentration across the whole sample population; The Relative Standard Deviation (RSD) for the two repeat-injection Quality Control patients (8 reps per patient evenly dispersed across the experimental run) – an RSD of <20% is considered acceptable; The Biological Signal to Noise ratio (S/N) in decibels (dB) – this gives an indication of biological information content, calculated using the following equation: 20 log (RMSsample/RMSQC), where RMS = Root Mean Squared amplitude of the mean centred data. A S/N >15 dB indicates excellent information content. (DOC) [file pone.0050520.s005.doc]

Table S1: List of quantified metabolites. Included for each metabolite: The recommended limit of detection for the specified platform; percentage of missing values (i.e. metabolite concentration below the measurable limit); mean serum concentration across the whole sample population; The Relative Standard Deviation (RSD) for the two repeat-injection Quality Control patients (8 reps per patient evenly dispersed across the experimental run) – an RSD of < 20% is considered acceptable; The Biological Signal to Noise ratio (S/N) in decibels (dB) – this gives an indication of biological information content, calculated using the following equation: 20 log (RMS_sample_/RMS_QC_), where RMS = Root Mean Squared amplitude of the mean centred data. A S/N > 15dB indicates excellent information content.

| idx | Name | Code | Class | Limit of detection (μM) | %age missing | Mean conc. (μM) | %RSD QC1 | %RSD QC2 | Biological S/N dB‡ |
| --- | --- | --- | --- | --- | --- | --- | --- | --- | --- |
| 1 | Carnitine | C0 | Acylcarnitines | 4 | 0 | 14.831 | 3 | 2 | 20.9 |
| 2 | Decenoylcarnitine | C10:1 | Acylcarnitines | 0.12 | 0 | 0.164 | 3 | 4 | 15.5 |
| 3 | Dodecanoylcarnitine | C12 | Acylcarnitines | 0.057 | 0 | 0.081 | 13 | 5 | 20.8 |
| 4 | Dodecanoylcarnitine | C12:1 | Acylcarnitines | 0.2 | 0 | 0.234 | 3 | 3 | 19.2 |
| 5 | Tetradecanoylcarnitine | C14 | Acylcarnitines | 0.03 | 0 | 0.064 | 9 | 13 | 22.0 |
| 6 | cis-5-Tetradecenoylcarnitine | C14:1 | Acylcarnitines | 0.015 | 0 | 0.093 | 6 | 6 | 25.5 |
| 7 | 3, 5-Tetradecadiencarnitine | C14:2 | Acylcarnitines | 0.012 | 0 | 0.020 | 14 | 10 | 16.9 |
| 8 | L-Palmitoylcarnitine | C16 | Acylcarnitines | 0.018 | 0 | 0.158 | 6 | 8 | 30.3 |
| 9 | Hexadecenoylcarnitine | C16:1 | Acylcarnitines | 0.06 | 0 | 0.052 | 24 | 22 | 14.8 |
| 10 | Stearoylcarnitine | C18 | Acylcarnitines | 0.02 | 0 | 0.029 | 3 | 15 | 29.5 |
| 11 | Oleoylcarnitine | C18:1 | Acylcarnitines | 0.04 | 0 | 0.098 | 7 | 8 | 26.1 |
| 12 | Linoelaidyl carnitine | C18:2 | Acylcarnitines | 0.009 | 0 | 0.046 | 9 | 11 | 24.4 |
| 13 | L-Acetylcarnitine | C2 | Acylcarnitines | 0.15 | 0 | 5.630 | 1 | 5 | 36.8 |
| 14 | Propionylcarnitine | C3 | Acylcarnitines | 0.08 | 0 | 0.335 | 5 | 3 | 16.1 |
| 15 | Hydroxybutyrylcarnitine (Malonylcarnitine) | C3-DC (C4-OH) | Acylcarnitines | 0.09 | 0 | 0.101 | 4 | 9 | 22.6 |
| 16 | Hydroxypropionylcarnitine | C3-OH | Acylcarnitines | 0.05 | 0 | 0.066 | 8 | 10 | 10.0 |
| 17 | Butyrylcarnitine | C4 | Acylcarnitines | 0.03 | 0 | 0.169 | 4 | 8 | 26.4 |
| 18 | Valerylcarnitine | C5 | Acylcarnitines | 0.04 | 0 | 0.133 | 4 | 6 | 26.7 |
| 19 | Hexanoylcarnitine (Fumarylcarnitine) | C6 (C4:1-DC) | Acylcarnitines | 0.08 | 0 | 0.094 | 8 | 12 | 18.5 |
| 20 | PC aa C28:1 | PC aa C28:1 | Glycerophospholipids | 0.04 | 0 | 0.997 | 4 | 5 | 23.2 |
| 21 | PC aa C30:0 | PC aa C30:0 | Glycerophospholipids | 0.2 | 0 | 3.862 | 4 | 3 | 23.0 |
| 22 | PC aa C30:2 | PC aa C30:2 | Glycerophospholipids | 0.006 | 30 | 0.045 | 12 | 14 | 21.1 |
| 23 | PC aa C32:0 | PC aa C32:0 | Glycerophospholipids | 0.04 | 0 | 16.211 | 2 | 4 | 30.6 |
| 24 | PC aa C32:1 | PC aa C32:1 | Glycerophospholipids | 0.06 | 0 | 17.046 | 4 | 4 | 25.7 |
| 25 | PC aa C32:2 | PC aa C32:2 | Glycerophospholipids | 0.03 | 0 | 0.618 | 9 | 5 | 15.9 |
| 26 | PC aa C32:3 | PC aa C32:3 | Glycerophospholipids | 0.008 | 0 | 0.184 | 4 | 4 | 21.9 |
| 27 | PC aa C34:1 | PC aa C34:1 | Glycerophospholipids | 0.06 | 0 | 137.798 | 34 | 4 | 1.9 |
| 28 | PC aa C34:2 | PC aa C34:2 | Glycerophospholipids | 0.1 | 0 | 111.237 | 4 | 4 | 24.1 |
| 29 | PC aa C34:3 | PC aa C34:3 | Glycerophospholipids | 0.01 | 0 | 4.230 | 3 | 2 | 26.2 |
| 30 | PC aa C34:4 | PC aa C34:4 | Glycerophospholipids | 0.006 | 0 | 0.538 | 4 | 2 | 20.9 |
| 31 | PC aa C36:0 | PC aa C36:0 | Glycerophospholipids | 0.2 | 0 | 2.171 | 4 | 3 | 21.7 |
| 32 | PC aa C36:1 | PC aa C36:1 | Glycerophospholipids | 0.03 | 0 | 26.704 | 6 | 6 | 19.5 |
| 33 | PC aa C36:2 | PC aa C36:2 | Glycerophospholipids | 0.15 | 0 | 59.199 | 4 | 6 | 23.9 |
| 34 | PC aa C36:3 | PC aa C36:3 | Glycerophospholipids | 0.04 | 0 | 73.586 | 3 | 6 | 23.2 |
| 35 | PC aa C36:4 | PC aa C36:4 | Glycerophospholipids | 0.04 | 0 | 158.228 | 3 | 6 | 24.8 |
| 36 | PC aa C36:5 | PC aa C36:5 | Glycerophospholipids | 0.01 | 0 | 6.951 | 5 | 1 | 25.3 |
| 37 | PC aa C36:6 | PC aa C36:6 | Glycerophospholipids | 0.015 | 0 | 0.315 | 3 | 4 | 23.1 |
| 38 | PC aa C38:0 | PC aa C38:0 | Glycerophospholipids | 0.2 | 0 | 1.841 | 6 | 5 | 23.4 |
| 39 | PC aa C38:1 | PC aa C38:1 | Glycerophospholipids | 0.08 | 4 | 0.325 | 6 | 2 | 20.0 |
| 40 | PC aa C38:3 | PC aa C38:3 | Glycerophospholipids | 0.04 | 0 | 42.417 | 8 | 7 | 14.8 |
| 41 | PC aa C38:4 | PC aa C38:4 | Glycerophospholipids | 0.03 | 0 | 105.445 | 3 | 4 | 24.4 |
| 42 | PC aa C38:5 | PC aa C38:5 | Glycerophospholipids | 0.015 | 0 | 28.252 | 9 | 7 | 13.4 |
| 43 | PC aa C38:6 | PC aa C38:6 | Glycerophospholipids | 0.02 | 0 | 78.864 | 3 | 4 | 23.1 |
| 44 | PC aa C40:2 | PC aa C40:2 | Glycerophospholipids | 0.02 | 0 | 0.182 | 9 | 3 | 20.3 |
| 45 | PC aa C40:3 | PC aa C40:3 | Glycerophospholipids | 0.006 | 0 | 0.569 | 6 | 2 | 19.4 |
| 46 | PC aa C40:4 | PC aa C40:4 | Glycerophospholipids | 0.01 | 0 | 3.363 | 4 | 3 | 22.6 |
| 47 | PC aa C40:5 | PC aa C40:5 | Glycerophospholipids | 0.04 | 0 | 7.093 | 2 | 2 | 26.8 |
| 48 | PC aa C40:6 | PC aa C40:6 | Glycerophospholipids | 1.2 | 0 | 31.169 | 6 | 4 | 18.5 |
| 49 | PC aa C42:0 | PC aa C42:0 | Glycerophospholipids | 0.05 | 0 | 1.640 | 4 | 1 | 61.4 |
| 50 | PC aa C42:1 | PC aa C42:1 | Glycerophospholipids | 0.008 | 0 | 0.302 | 8 | 2 | 15.2 |
| 51 | PC aa C42:2 | PC aa C42:2 | Glycerophospholipids | 0.006 | 0 | 0.125 | 10 | 5 | 15.5 |
| 52 | PC aa C42:4 | PC aa C42:4 | Glycerophospholipids | 0.006 | 0 | 0.244 | 6 | 4 | 18.6 |
| 53 | PC aa C42:5 | PC aa C42:5 | Glycerophospholipids | 0.05 | 0 | 0.343 | 4 | 3 | 22.7 |
| 54 | PC aa C42:6 | PC aa C42:6 | Glycerophospholipids | 0.3 | 0 | 0.467 | 4 | 1 | 16.9 |
| 55 | PC ae C30:0 | PC ae C30:0 | Glycerophospholipids | 0.15 | 0 | 0.334 | 5 | 4 | 18.6 |
| 56 | PC ae C30:1 | PC ae C30:1 | Glycerophospholipids | 0.02 | 0 | 0.101 | 4 | 8 | 24.2 |
| 57 | PC ae C32:1 | PC ae C32:1 | Glycerophospholipids | 0.009 | 0 | 2.475 | 4 | 2 | 25.6 |
| 58 | PC ae C32:2 | PC ae C32:2 | Glycerophospholipids | 0.02 | 0 | 0.515 | 4 | 1 | 23.1 |
| 59 | PC ae C34:0 | PC ae C34:0 | Glycerophospholipids | 0.017 | 0 | 1.305 | 5 | 3 | 22.4 |
| 60 | PC ae C34:1 | PC ae C34:1 | Glycerophospholipids | 0.012 | 0 | 5.442 | 3 | 1 | 26.4 |
| 61 | PC ae C34:2 | PC ae C34:2 | Glycerophospholipids | 0.01 | 0 | 3.495 | 6 | 2 | 21.8 |
| 62 | PC ae C34:3 | PC ae C34:3 | Glycerophospholipids | 0.015 | 0 | 1.315 | 4 | 2 | 28.4 |
| 63 | PC ae C36:0 | PC ae C36:0 | Glycerophospholipids | 0.12 | 0 | 0.717 | 4 | 1 | 22.6 |
| 64 | PC ae C36:1 | PC ae C36:1 | Glycerophospholipids | 0.03 | 0 | 2.924 | 5 | 2 | 21.8 |
| 65 | PC ae C36:2 | PC ae C36:2 | Glycerophospholipids | 0.01 | 0 | 2.970 | 5 | 2 | 22.2 |
| 66 | PC ae C36:3 | PC ae C36:3 | Glycerophospholipids | 0.007 | 0 | 2.150 | 5 | 3 | 22.1 |
| 67 | PC ae C36:4 | PC ae C36:4 | Glycerophospholipids | 0.013 | 0 | 10.071 | 4 | 1 | 23.7 |
| 68 | PC ae C36:5 | PC ae C36:5 | Glycerophospholipids | 0.012 | 0 | 6.705 | 3 | 1 | 27.2 |
| 69 | PC ae C38:0 | PC ae C38:0 | Glycerophospholipids | 0.066 | 0 | 1.403 | 6 | 4 | 16.4 |
| 70 | PC ae C38:1 | PC ae C38:1 | Glycerophospholipids | 0.015 | 0 | 0.357 | 9 | 1 | 22.6 |
| 71 | PC ae C38:2 | PC ae C38:2 | Glycerophospholipids | 0.018 | 0 | 0.712 | 4 | 1 | 29.7 |
| 72 | PC ae C38:3 | PC ae C38:3 | Glycerophospholipids | 0.01 | 0 | 2.417 | 4 | 1 | 23.7 |
| 73 | PC ae C38:4 | PC ae C38:4 | Glycerophospholipids | 0.015 | 0 | 9.505 | 3 | 1 | 24.5 |
| 74 | PC ae C38:5 | PC ae C38:5 | Glycerophospholipids | 0.01 | 0 | 8.340 | 2 | 2 | 27.9 |
| 75 | PC ae C38:6 | PC ae C38:6 | Glycerophospholipids | 0.03 | 0 | 5.024 | 5 | 1 | 42.5 |
| 76 | PC ae C40:1 | PC ae C40:1 | Glycerophospholipids | 0.06 | 0 | 0.646 | 4 | 2 | 24.0 |
| 77 | PC ae C40:2 | PC ae C40:2 | Glycerophospholipids | 0.01 | 0 | 0.813 | 3 | 1 | 25.3 |
| 78 | PC ae C40:3 | PC ae C40:3 | Glycerophospholipids | 0.015 | 0 | 0.755 | 3 | 1 | 28.4 |
| 79 | PC ae C40:4 | PC ae C40:4 | Glycerophospholipids | 0.1 | 0 | 1.913 | 7 | 2 | 16.4 |
| 80 | PC ae C40:5 | PC ae C40:5 | Glycerophospholipids | 0.006 | 0 | 1.694 | 6 | 3 | 19.2 |
| 81 | PC ae C40:6 | PC ae C40:6 | Glycerophospholipids | 0.025 | 0 | 2.669 | 4 | 2 | 23.9 |
| 82 | PC ae C42:1 | PC ae C42:1 | Glycerophospholipids | 0.03 | 0 | 0.267 | 4 | 2 | 23.9 |
| 83 | PC ae C42:2 | PC ae C42:2 | Glycerophospholipids | 0.006 | 0 | 0.251 | 5 | 1 | 23.1 |
| 84 | PC ae C42:3 | PC ae C42:3 | Glycerophospholipids | 0.006 | 0 | 0.302 | 4 | 1 | 22.7 |
| 85 | PC ae C42:4 | PC ae C42:4 | Glycerophospholipids | 0.3 | 1 | 0.533 | 7 | 1 | 17.6 |
| 86 | PC ae C42:5 | PC ae C42:5 | Glycerophospholipids | 1.3 | 0 | 1.321 | 15 | 16 | 5.7 |
| 87 | PC ae C44:3 | PC ae C44:3 | Glycerophospholipids | 0.006 | 0 | 0.088 | 15 | 2 | 17.6 |
| 88 | PC ae C44:4 | PC ae C44:4 | Glycerophospholipids | 0.01 | 0 | 0.302 | 5 | 2 | 19.5 |
| 89 | PC ae C44:5 | PC ae C44:5 | Glycerophospholipids | 0.02 | 0 | 1.072 | 3 | 1 | 25.2 |
| 90 | PC ae C44:6 | PC ae C44:6 | Glycerophospholipids | 0.09 | 0 | 0.866 | 3 | 1 | 22.7 |
| 91 | lysoPC a C16:0 | lysoPC a C16:0 | Glycerophospholipids | 0.12 | 0 | 62.264 | 6 | 3 | 14.5 |
| 92 | lysoPC a C16:1 | lysoPC a C16:1 | Glycerophospholipids | 0.07 | 0 | 4.499 | 2 | 1 | 28.3 |
| 93 | lysoPC a C17:0 | lysoPC a C17:0 | Glycerophospholipids | 0.05 | 0 | 0.759 | 4 | 1 | 19.6 |
| 94 | lysoPC a C18:0 | lysoPC a C18:0 | Glycerophospholipids | 0.05 | 0 | 10.862 | 7 | 4 | 15.5 |
| 95 | lysoPC a C18:1 | lysoPC a C18:1 | Glycerophospholipids | 0.1 | 0 | 13.371 | 7 | 2 | 15.5 |
| 96 | lysoPC a C18:2 | lysoPC a C18:2 | Glycerophospholipids | 0.1 | 0 | 13.477 | 5 | 2 | 18.7 |
| 97 | lysoPC a C20:3 | lysoPC a C20:3 | Glycerophospholipids | 0.2 | 0 | 3.540 | 6 | 3 | 19.8 |
| 98 | lysoPC a C20:4 | lysoPC a C20:4 | Glycerophospholipids | 0.02 | 0 | 14.436 | 4 | 4 | 21.4 |
| 99 | lysoPC a C28:0 | lysoPC a C28:0 | Glycerophospholipids | 0.3 | 0 | 0.426 | 3 | 0 | 21.8 |
| 100 | lysoPC a C28:1 | lysoPC a C28:1 | Glycerophospholipids | 0.15 | 0 | 0.344 | 7 | 1 | 12.7 |
| 101 | SM (OH) C14:1 | SM (OH) C14:1 | Sphingolipids | 0.025 | 0 | 2.096 | 4 | 1 | 22.1 |
| 102 | SM (OH) C16:1 | SM (OH) C16:1 | Sphingolipids | 0.012 | 0 | 1.612 | 4 | 19 | 20.4 |
| 103 | SM (OH) C22:1 | SM (OH) C22:1 | Sphingolipids | 0.015 | 0 | 3.933 | 4 | 1 | 21.8 |
| 104 | SM (OH) C22:2 | SM (OH) C22:2 | Sphingolipids | 0.01 | 0 | 3.657 | 6 | 1 | 18.9 |
| 105 | SM (OH) C24:1 | SM (OH) C24:1 | Sphingolipids | 0.01 | 0 | 1.039 | 2 | 3 | 30.3 |
| 106 | SM C16:0 | SM C16:0 | Sphingolipids | 0.03 | 0 | 58.168 | 5 | 2 | 18.4 |
| 107 | SM C16:1 | SM C16:1 | Sphingolipids | 0.01 | 0 | 10.868 | 2 | 5 | 29.9 |
| 108 | SM C18:0 | SM C18:0 | Sphingolipids | 0.07 | 0 | 18.819 | 4 | 3 | 20.5 |
| 109 | SM C18:1 | SM C18:1 | Sphingolipids | 0.01 | 0 | 12.857 | 2 | 2 | 29.3 |
| 110 | SM C20:2 | SM C20:2 | Sphingolipids | 0.005 | 0 | 0.346 | 4 | 1 | 23.7 |
| 111 | SM C22:3 | SM C22:3 | Sphingolipids | 0.01 | 2 | 0.386 | 2 | 1 | 27.7 |
| 112 | SM C24:0 | SM C24:0 | Sphingolipids | 0.13 | 0 | 16.444 | 4 | 2 | 21.2 |
| 113 | SM C24:1 | SM C24:1 | Sphingolipids | 0.035 | 0 | 28.642 | 5 | 3 | 18.5 |
| 114 | SM C26:0 | SM C26:0 | Sphingolipids | 0.015 | 0 | 0.208 | 11 | 16 | 15.7 |
| 115 | SM C26:1 | SM C26:1 | Sphingolipids | 0.006 | 0 | 0.409 | 5 | 1 | 22.5 |
| 116 | Hexose | Hexose | Sugars | 20 | 0 | ####### | 4 | 1 | 19.5 |
| 117 | Alanine | Alanine | Aminoacids | 1.00 | 0 | 510.351 | 1 | 1 | 29.2 |
| 118 | Arginine | Arginine | Aminoacids | 0.50 | 0 | 93.056 | 2 | 2 | 33.9 |
| 119 | Asparagine | Asparagine | Aminoacids | 1.50 | 0 | 48.372 | 6 | 2 | 15.5 |
| 120 | Aspartate | Aspartate | Aminoacids | 1.50 | 0 | 29.100 | 2 | 5 | 25.3 |
| 121 | Citrulline | Citrulline | Aminoacids | 1.00 | 0 | 11.938 | 4 | 0 | 17.6 |
| 122 | Glutamine | Glutamine | Aminoacids | 1.50 | 0 | 476.532 | 1 | 1 | 28.2 |
| 123 | Glutamate | Glutamate | Aminoacids | 2.00 | 0 | 92.707 | 2 | 3 | 30.8 |
| 124 | Glycine | Glycine | Aminoacids | 0.50 | 0 | 264.721 | 1 | 1 | 26.8 |
| 125 | Histidine | Histidine | Aminoacids | 0.50 | 0 | 109.875 | 1 | 2 | 30.8 |
| 126 | Isoleucine | Isoleucine | Aminoacids | 1.50 | 0 | 72.830 | 1 | 2 | 27.2 |
| 127 | Leucine | Leucine | Aminoacids | 1.50 | 0 | 139.475 | 2 | 3 | 25.5 |
| 128 | Lysine | Lysine | Aminoacids | 0.50 | 0 | 386.519 | 2 | 1 | 25.0 |
| 129 | Methionine | Methionine | Aminoacids | 0.10 | 0 | 30.247 | 2 | 3 | 23.5 |
| 130 | Ornithine | Ornithine | Aminoacids | 0.50 | 0 | 78.026 | 2 | 6 | 28.5 |
| 131 | Phenylalanine | Phenylalanine | Aminoacids | 0.10 | 0 | 83.591 | 1 | 2 | 29.2 |
| 132 | Proline | Proline | Aminoacids | 1.00 | 0 | 157.955 | 2 | 2 | 21.7 |
| 133 | Serine | Serine | Aminoacids | 1.00 | 0 | 133.705 | 3 | 2 | 17.0 |
| 134 | Threonine | Threonine | Aminoacids | 0.50 | 0 | 212.669 | 1 | 1 | 28.1 |
| 135 | Tryptophan | Tryptophan | Aminoacids | 0.50 | 0 | 77.441 | 2 | 2 | 28.8 |
| 136 | Tyrosine | Tyrosine | Aminoacids | 0.50 | 0 | 66.433 | 2 | 2 | 24.0 |
| 137 | Valine | Valine | Aminoacids | 0.50 | 0 | 210.500 | 1 | 2 | 27.7 |
| 138 | Acetylornithine(Ac-Orn) | Ac-Orn | Biogenic Amines | 0.15 | 0 | 0.423 | 4 | 2 | 35.2 |
| 139 | Asymmetric dimethylarginine (ADMA) | ADMA | Biogenic Amines | 0.08 | 0 | 1.178 | 5 | 1 | 11.9 |
| 140 | alpha-Aminoadipic acid(AAA) | AAA | Biogenic Amines | 0.30 | 0 | 1.246 | 1 | 3 | 65.9 |
| 141 | Carnosine | Carnosine | Biogenic Amines | 0.10 | 0 | 0.620 | 0 | 1 | 41.1 |
| 142 | Creatinine | Creatinine | Creatinine | 1.00 | 0 | 71.768 | 3 | 2 | 19.7 |
| 143 | Histamine | Histamine | Biogenic Amines | 0.30 | 0 | 0.470 | 1 | 1 | 25.9 |
| 144 | Kynurenine | Kynurenine | Biogenic Amines | 0.30 | 0 | 6.229 | 2 | 1 | 25.8 |
| 145 | Methioninesulfoxide(Met-SO) | Met-SO | Biogenic Amines | 0.30 | 0 | 0.681 | 1 | 1 | 24.4 |
| 146 | Putrescine | Putrescine | Biogenic Amines | 0.03 | 3 | 0.304 | 2 | 2 | 39.7 |
| 147 | Spermidine | Spermidine | Biogenic Amines | 0.08 | 0 | 1.235 | 1 | 1 | 50.5 |
| 148 | Taurine | Taurine | Biogenic Amines | 0.50 | 1 | 194.897 | 13 | 37 | 7.5 |
