# Supplementary material for: The Metabolomic Profile of Umbilical Cord Blood in Neonatal Hypoxic Ischaemic Encephalopathy
Source: PLoS One. 2012 Dec 5;7(12):e50520. doi: 10.1371/journal.pone.0050520 (PMC3515614; doi:10.1371/journal.pone.0050520)
Supplement: Table S2 — Table of the parameter estimates generated by the covariate adjusted ROC analysis (using non-parametric bootstrap AUC estimation and a linear correction model). The associated corrected area under the ROC curve (AUC) was 0.93 (95% CI: 0.81–0.99); this was not signifiacntly different to the uncorrected ROC curve. T-tests estimating the contribution of each clinical variable to the linear correction model also indicate that each clinical variable had no significant `influence on the corrected ROC curve. Thus we conclude that these potential confounders had neither a significant positive nor negative impact on the metabolite biomarker signature. (DOC) [file pone.0050520.s006.doc]

Table S2: Table of the parameter estimates generated by the covariate adjusted ROC analysis (using non-parametric bootstrap AUC estimation and a linear correction model). The associated corrected area under the ROC curve (AUC) was 0.93 (95% CI: 0.81-0.99); this was not signifiacntly different to the uncorrected ROC curve. T-tests estimating the contribution of each clinical variable to the linear correction model also indicate that each clinical variable had no significant `influence on the corrected ROC curve. Thus we conclude that these potential confounders had neither a significant positive nor negative impact on the metabolite biomarker signature.

| **Clinical Variable** | **β** | **t-score** | **P>|t|** | **β - 95% Conf.** | |
| --- | --- | --- | --- | --- | --- |
| Gender (Male) | -0.0037 | -0.1 | 0.918 | -0.0751 | 0.0677 |
| Gestational Age | -0.0193 | -1.22 | 0.226 | -0.0507 | 0.0122 |
| Birth Weight Centile | -0.0002 | -0.39 | 0.698 | -0.0013 | 0.0008 |
| Maternal Age | 0.0028 | 0.08 | 0.939 | -0.0680 | 0.0735 |
| Maternal BMI | -0.0170 | -1.08 | 0.281 | -0.0481 | 0.0142 |
| Method of Delivery (Elective) | -0.0003 | -0.62 | 0.539 | -0.0014 | 0.0007 |
| Method of Delivery (Emergency) | -0.0017 | -0.51 | 0.609 | -0.0081 | 0.0048 |
| Method of Delivery (Instrumental) | 0.0045 | 1.14 | 0.258 | -0.0034 | 0.0125 |
| Constant offset β_0_ | 0.7300 | 1.15 | 0.255 | -0.5355 | 1.9955 |
